# Thirst in critical patients and its associated factors

**DOI:** 10.1590/0034-7167-2024-0064

**Published:** 2025-03-10

**Authors:** Laura Vicentim Berbert, Isadora Pierotti, Leonel Alves do Nascimento, Isabela Bossi Faleiros, Meiriane Pizani Scobare de Oliveira, Rafael Alexandre Biz, Rafaela Vieira Jorge, Lígia Fahl Fonseca

**Affiliations:** I Universidade Estadual de Londrina. Londrina, Paraná, Brazil

**Keywords:** Thirst, Intensive Care Units, Nursing Care, Patient Comfort, Critical Care, Sed, Unidades de Cuidados Intensivos, Atención de Enfermería, Comodidad del Paciente, Cuidados Críticos

## Abstract

**Objectives:**

to assess prevalence, intensity, discomfort, defining characteristics of thirst and signs of oral mucosa hydration in Intensive Care Unit patients.

**Methods:**

quantitative and analytical study, carried out in a tertiary hospital in six of the seven Intensive Care Units, with a sample of 60 patients. Variables related to thirst were analyzed according to their nature.

**Results:**

prevalence of thirst was 76.7%, with a mean intensity of 7.2. The main Objective Oral Mucosa Scale score was 2 (61.7%), corresponding to dry lips and moist mouth. Prevalent defining characteristics were thick saliva (80%), constant swallowing of saliva (76.7%), desire to drink water (75%), dry lips (73.3%) and dry throat (70%). Positive correlations were identified between scales and thirst intensity, water restriction and hospitalization duration.

**Conclusions:**

the statistically significant correlations reflect the complexity and multifactorial nature of thirst, demonstrating the need to use identification and measurement instruments in the critical population.

## RESUMEN

**Objetivos:**

evaluar prevalencia, intensidad, malestar, características definitorias de la sed y signos de hidratación de la mucosa oral en pacientes de Unidad de Cuidados Intensivos.

**Métodos:**

estudio cuantitativo y analítico, realizado en un hospital terciario en seis de las siete Unidades de Cuidados Intensivos, con una muestra de 60 pacientes. Se analizaron las variables relacionadas con la sede según su naturaliza.

**Resultados:**

la prevalencia de sed fue de 76,7%, intensidad promedio de 7,2. El puntaje principal de la *Objective Oral Mucosa Scale* fue 2 (61,7%), correspondiente a labios secos y boca húmeda. Las características definitorias predominantes fueron saliva espesa (80%), deglución constante de saliva (76,7%), deseo de beber agua (75%), labios secos (73,3%) y garganta seca (70%). Se identificaron correlaciones positivas entre las escalas y la intensidad de la sed, el tiempo de restricción de agua y la hospitalización.

**Conclusiones:**

las correlaciones estadísticamente significativas reflejan la complejidad y naturaleza multifactorial de la sed, demostrando la necesidad de utilizar instrumentos de identificación y medición en la población crítica.

## INTRODUCTION

Thirst defined as the need to drink water, arises from the body’s physiological need to restore its hydroelectrolytic balance. Physiologically, it can be divided into homeostatic, which integrates organic and osmotic functions, with a direct influence on blood volume, and non-homeostatic, which addresses aspects related to thirst, such as characteristics of water and food intake, individual lifestyle habits and, in particular, stimuli triggered by oral cavity dryness^(1)^.

In hospital settings, several factors are considered stressors and can result in the genesis of thirst. These include surgical procedures that require water restriction, anesthetic modalities adopted, orotracheal intubation, risk of hemorrhage, among others^(2)^, such as the process of becoming ill and the insecurities that permeate this moment^(3)^.

This discomfort also affects critically ill patients. In Intensive Care Units (ICU), thirst is highly prevalent, reaching 68.9%, and is categorized as mild (30.9%), moderate (42.9%) and severe (26.2%) ^(4)^. Specific studies for this population are still recent, and attention to this discomfort should be part of care plan^(5)^, since patients in this group are susceptible to aggravating factors, such as greater fluid imbalance, drug therapies marked mainly by the use of anticholinergics^(6)^, opioids^(7,8,9)^, antihypertensives^(6)^, antidepressants^(10)^, mechanical ventilation (MV)^(5)^ and water and food restrictions^(11)^.

The combination of endotracheal devices and sedative medications, common procedures in ICUs, contributes to many patients becoming unconscious and unable to spontaneously verbalize their thirst and the discomfort it causes^(12)^. In critically ill patients, therefore, the self-report of a subjective symptom, considered the gold standard for its identification and measurement, is impaired. The low frequency of verbalization of thirst suggests the need for special attention from the health team, especially nurses, to the defining characteristics of thirst and encouragement of patients to verbalize it so that the symptom can be correctly identified and managed. Healthcare professionals should seekto alleviate discomforts beyond those already known in ICUs as well as make a formal and legal record of them so that there is continuity of care^(5,6)^. However, the number of tools created exclusively for thirst management in the critically ill population is scarce, and they are often not validated by specialists^(7)^.

In the search for awareness and correct management of thirst by the multidisciplinary team, the need arose to develop a proposal for a diagnosis to be included in diagnostic taxonomies, such as NANDA-I, originally developed as a diagnosis of perioperative thirst, aimed mainly at the surgical population. Structured in the instrument standards, the diagnostic proposal presents the main etiological factors of thirst and its related factors, highlighting the defining characteristics as follows: dry lips; desire to drink water; constant swallowing of saliva; dry throat; thick saliva; thick tongue; bad taste in the mouth; and caregiver report. The assessment of the defining characteristics of thirst allows the identification of signs by the nursing team to complement, and can be used with critically ill patients in the ICU^(13)^.

The Objective Oral Mucosa Scale is a Chinese photographic scale developed to assess oral cavity dehydration. Using this scale made it possible to recognize xerostomia as the most prevalent thirst characteristic in the critically ill population, proving to be a useful instrument in the use of previously tested and approved thirst relief intervention packages^(14)^.

Thirst is complex and multifactorial^(15)^ so its assessment should not be limited to its intensity alone; the discomfort it causes must also be considered. In this regard, the Perioperative Thirst Discomfort Scale (EDESP - *Escala de Desconforto da Sede Perioperatória*) was developed, a Likert-type scale that assesses seven related attributes, with high levels of reliability and fidelity^(16)^.

Considering the high number of ICU patients who experience thirst, the scarcity of studies conducted with this population, and the difficulty in managing thirst in this group of patients due to their specificities and inherent criticality, the present study is necessary to better assess thirst and its defining characteristics. Additionally, scales that allow easy identification of characteristic signs of thirst can be useful tools for health teams in their intentional care of this symptom.

## OBJECTIVES

To assess prevalence, intensity, discomfort, defining characteristics of thirst and signs of oral mucosa hydration in ICU patients and related factors.

## METHODS

### Ethical aspects

This study is part of a larger project entitled “Effects of mentholated popsicles on vasopressin secretion, osmolarity, intensity, thirst discomfort and oral dehydration: Randomized Clinical Trial”, approved by the Research Ethics Committee. To carry out this research, it was necessary to make an amendment to the previous opinion. The study was conducted in accordance with national and international ethics guidelines and was approved by the *Universidade Estadual de Londrina* Research Ethics Committee, whose opinion is attached to this submission. The Informed Consent Form was obtained from all individuals involved in the study in writing.

### Study design, period and location

A quantitative, observational, cross-sectional, and analytical study was carried out in the ICUs of a tertiary university hospital in the state of Paraná. This is a public hospital available to the Brazilian Health System (SUS - *Sistema Único de Saúde*), which covers teaching and research areas, with 419 beds. It has seven ICUs for adult care, with 64 specialized beds, covering surgical, clinical, isolation, long-stay, and highly dependent patients, and one of the units is specialized in burns. Data collection took place in six of the seven ICUs in the hospital, excluding the Burn Treatment Unit, between September and December 2022. For this study, the STrengthening the Reporting of OBservational studies in Epidemiology (STROBE) was used to guide the methodology.

### Population or sample; inclusion and exclusion criteria

The sample was non-probabilistic, totaling 60 patients, and followed the inclusion criteria that included being over 18 years of age, being oriented in time and space, asking patients directly “What is your name? Where are you right now? What day is it today?”, according to the Montreal Cognitive Assessment (MoCa) protocol^(17)^, patients on oral fluid restriction and the ability to verbalize thirst spontaneously or when asked. The exclusion criteria included patients using MV and/or tracheostomized, due to the use of medications that prevent full orientation in time and space.

### Study protocol

A script was used with aspects about hospitalization, presence and justification of oral fluid restriction and use of medications. Thirst presence and intensity were assessed using the Verbal Numerical Scale (VNS), with a range from zero to ten, with zero being no thirst and ten being the worst thirst. Thirst discomforts were assessed using the EDESP, which assessed attributes through direct observation or through patient report, such as dry mouth, dry lips, thick tongue, thick saliva, dry throat, bad taste in the mouth and desire to drink water, with a maximum score of 14 points, this being the highest degree of thirst presented by patient. This scale presents reliability and fidelity indices with Cronbach’s alpha of 0.91^(16)^. The previously mentioned defining characteristics of thirst were also assessed^(13)^.

Oral mucosal dehydration was identified using the Objective Oral Mucosa Scale, which has four scores: 1 = moist lips and mouth; 2 = dry lips and moist mouth; 3 = dry lips and mouth; and 4 = cracked lips and dry mouth. Its reliability and interobserver correlation coefficient was 0.89^(14)^.

Clinical data were extracted from the information present in the medical records. Finally, the Objective Oral Mucosa Scale, positioned next to patients’ oral cavity, was compared, visually identifying patients and recording the score on the research instrument.

The researcher elaborated the research script, which was submitted for apparent analysis by two experts on the topic of thirst. A pilot test was applied with five patients; there was no need to make changes to the instrument; and the data were not included in the sample.

### Analysis of results, and statistics

Dependent variables of this study were thirst presence and intensity, hydration signs of the Objective Oral Mucosa Scale and defining characteristics of thirst. Independent variables were sociodemographic and clinical data, such as sex, age, length of ICU stay, reason for hospitalization, duration of oral fluid restriction, use of medications and previous use of MV devices.

The data were structured in a Microsoft Excel 2019® spreadsheet, validated by double entry and analyzed with the aid of the Jamovi Version 2.3 statistical program. For data analysis, descriptive analysis was used, with variables presented in absolute and relative frequencies as well as measures of central tendency and dispersion. The Shapiro-Wilk test was applied to assess whether the data adhered to normality. For correlation, the Spearman test was used for numerical variables, and the chi-square association test (X^2^), for categorical variables, both with a significance level of 0.05. The tests that used Spearman had their correlation strengths interpreted as follows: weak or small (values observed between 0.1 and 0.3 or -0.1 and -0.3); moderate or medium (values observed between 0.3 and 0.5 or -0.3 and -0.5); and strong (observed values between 0.5 and 1.0 or -0.5 and -1.0)^(18)^.

## RESULTS

Study participants were predominantly male (65%) and had a mean age of 54.6 years (±16.6). The most prevalent clinical area was cardiology (36.7%), and the mean hospital stay was 11.2 days (±22.3). The mean time of oral fluid restriction was 37.1 hours (±79.2), with a minimum of one hour and a maximum of 432 hours. During hospitalization, the use of opioid medications prevailed (53.3%). Table 1 shows participant characterization data.

**Table 1:** Sociodemographic and clinical data of data collection participants (N=60), Londrina, Paraná, Brazil, 2022

Variables		n	%
Sex			
Male		39	65.0
Female		21	35.0
Clinic			
Cardiology		22	36.7
Neurosurgery		08	13.3
Gastroenterology		07	11.7
Digestive system surgery		05	08.3
Others		18	30.1
Nutritional intake devices			
Nasoenteral catheter		06	10.0
Parenteral nutrition		01	01.7
Complete fasting without the need for devices		53	88.3
Reason for the need for oral water restriction				
Exams		07	11.7
Pre-operative		23	38.3
Post-operative		21	35.0
Others		09	15.0
Medications in use			
Opioids		32	53.3
Antidepressants		14	23.3
Anticholinergics		06	10.0
Antihypertensives		28	46.7
Use of prior mechanical ventilation during hospitalization			
Yes		18	30.0
No		42	70.0
**Continuous variables**	**A/lean (±)**	**Median (Minimum-Maximum)**	*p* value*
Age	54.6 (16.6)	59.0 (19-80)	0.015
Days of hospitalization	11.2 (22.3)	05.0 (01-157)	<0.001
Duration of oral fluid restriction (hours)	37.1 (79.2)	12 (01-432)	<0.001

* *p value referring to the Shapiro-Wilk normality test.*

Thirst prevalence was 76.7% (46/60). The mean thirst intensity was 7.2, measured by the VNS, with a score ranging from zero to ten, thus showing high intensity. Regarding the EDESP, it has a score ranging from zero to 14, with the mean score for discomfort from thirst being assessed as 7.3 points, reflecting that thirst, in addition to being intense, is also highly uncomfortable for critically ill patients. The main score observed by the Objective Oral Mucosa Scale was 2 (61.7%), corresponding to dry lips and moist mouth, with a score of 4 being the only one not identified. Table 2 shows thirst presence, intensity, discomfort from thirst, measured by the EDESP and Objective Oral Mucosa Scale score.

**Table 2:** Intensity, thirst discomfort (EDESP) and Objective Oral Mucosa Scale in data collection participants (N=60), Londrina, Paraná, Brazil, 2022

Variables	Mean (±)	Median (Minimum-Maximum)	p value*
Thirst intensity	07.2 (02.3)	08 (2-10)	0.020
EDESP Score**	07.4 (04.2)	07 (0-14)	<0.001
Objective Oral Mucosa Scale	01.9 (0.6)	02 (1-3)	
Objective Oral Mucosa Scale	**n**	**%**	
Score 1	15	25.0	
Score 2	37	61.7	
Score 3	08	13.3	
Score 4	00	0.0	
**EDESP**	**Not bothered at all n (%)**	**A little bothered n (%)**	**Very bothered n (%)**
My mouth is dry	17 (28.3)	18 (30.0)	25 (41.7)
My lips are dry	21 (35.0)	13 (21.7)	26 (43.3)
My tongue is thick	28 (46.7)	12 (20.0)	20 (33.3)
My saliva is thick	17 (28.3)	18 (30.0)	25 (41.7)
My throat is dry	21 (35.0)	14 (23.3)	25 (41.7)
I have a bad taste in my mouth	28 (46.7)	13 (21.7)	19 (31.7)
I have a desire to drink water	15 (25.0)	16 (26.7)	29 (48.3)

*p value referring to the Shapiro-Wilk normality test; **EDESP - Perioperative Thirst Discomfort Scale.

Among the defining characteristics of thirst, the most prevalent were thick saliva (80%), constant swallowing of saliva (76.7%), desire to drink water (75%), dry lips (73.3%) and dry throat (70%). All defining characteristics of thirst were present among patients and are presented in Table 3.

**Table 3:** Defining characteristics of the headquarters of data collection participants (N=60), Londrina, Paraná, Brazil, 2022

DEFINING CHARACTERISTICS	YES n (%)	NO n (%)
Dry throat	42 (70.0)	18 (30.0)
Dry lips	44 (73.3)	16 (26.7)
Thick saliva	48 (80.0)	12 (20.0)
Thick tongue	39 (65.0)	21 (35.0)
Constant swallowing of saliva	46 (76.7)	14 (23.3)
Desire to drink water	45 (75.0)	15 (25.0)
Bad taste in the mouth	33 (55.0)	27 (45.0)
Caregiver report	02 (3.3)	58 (96.7)

There were moderate positive correlations between: EDESP and Objective Oral Mucosa Scale; time of oral fluid restriction and EDESP; length of hospital stay and EDESP; thirst intensity and EDESP; and thirst intensity and Objective Oral Mucosa Scale. The correlations can be seen in Chart 1.

**Chart 1:** Spearman correlation coefficients (r_s_) between variables related to thirst (N=60), Londrina, Paraná, Brazil, 2022

Variables	EDESP*	Thirst intensity	Objective Oral Mucosa Scale	Oral water restriction	Days of hospitalization
EDESP	-				
Thirst intensity	r_s=_0.473 p<0.001	-			
*Objective Oral Mucosa Scale*	r_s=_0.364 p 0.004	r_s=_0.424 p 0.003	-		
Oral water restriction	r_s=_0.378 p 0.003	r_s=_0.303 p 0.041	r_s=_0.230 p 0.077	-	
Days of hospitalization	r_s=_0.332 p 0.009	r_s=_-0.029 p 0.846	r_s=_0.094 p 0.474	r_s=_-0.046 p 0.729	-

* *EDESP - Perioperative Thirst Discomfort Scale (Escala de Desconforto da Sede Perioperatória).*

The analysis of categorical variables (medications, use of MV and thirst presence) did not find significant data, as observed in Table 4.

**Table 4:** Categorical variables correlated with thirst presence, Londrina, Paraná, Brazil, 2022

Variables	X^2^	*p* value*
Opioid medications	3.29	0.070
Anticholinergic medications	0.839	0.360
Antidepressant medications	0.0284	0.866
Antihypertensive medications	2.28	0.131
Previous use of mechanical ventilation	3.23	0.072

* *chi-square test (X^2^) with continuity correction.*

## DISCUSSION

This study presents an overview of thirst assessment in critically ill patients and its associated factors within a complex scenario, that of ICU patients, a topic insufficiently explored in the literature. Nevertheless, thirst prevalence was assessed using a scale originally structured for patients in the immediate postoperative period, in addition to intensity, its defining characteristics and how these generate discomfort in critically ill patients. Moreover, assessment was complemented by a photographic scale focused on oral mucosa hydration, one of the main sources of thirst.

The field of intensive care commonly addresses populations of patients with delicate clinical conditions and who require long-term hospitalizations in specialized sectors, since therapeutic resources may include surgical approaches and use of MV^(5,8)^. The culture of prolonged fasting, a striking characteristic of the hospital where the research was developed, opposes the oral fluid restriction time recommended by the American Society of Anesthesiologists (ASA), which corresponds to up to two hours for clear liquids^(19)^ for surgical patients in the preoperative period.

Although the use of MV by patients in this study was low, oroand endotracheal devices were shown to be determinants for the genesis of thirst^(5)^. These attributes are considered predisposing factors for thirst, and this symptom is an important stressor, especially for patients with heart disease^(20)^.

Thirst is high and intense among critically ill patients, with a prevalence observed above 75%, expressed mainly by severe thirst in 44.9% of patients^(6)^. The high intensity of the symptom in the present study reinforces this population as being at high riskfor the development of thirst, due to numerous triggering factors, such as the use of opioids and diuretics, serum sodium and glucose levels, oral intake capacity, fasting, osmolarity and MV with oral cavity dryness^(4)^. Other studies show the role of administration of specialized drugs, devices and invasive approaches as predictors of thirst^(1,6,21)^. Thus, it is observed that both homeostatic thirst and non-homeostatic thirst were activated by pathophysiological and environmental mechanisms^(1)^.

Since thirst is characterized as intense discomfort that results in negative feelings for patients^(8,22)^, the nursing team must be intentional in identifying and managing discomfort correctly^(5)^. Strategies that trigger pre-absorptive satiety mechanisms through cold temperatures and menthol have proven effective in significantly alleviating thirst and discomfort in intensive care patients. Measures such as menthol sprays provide low-volume relief and, consequently, greater safety for critically ill patients on fluid restriction^(23)^.

Thirst can be identified by applying specific scales. The EDESP, originally designed to assist patients in the immediate postoperative period^(16)^, has proven to be a versatile scale. Capable of identifying several factors of discomfort, it is not limited to its target population, since the critical and surgical sectors share common aspects, such as fasting, medications, the need for MV and the complexity of their patients^(4,5)^. The use of this scale in the ICU yielded results parallel to those of research with surgical patients, presenting an mean discomfort intensity of 7.3 and the attribute “I have a desire to drink water” as the most uncomfortable^(24)^. The applicability of a scale aimed at discomfort in the routine assessment of ICU patients proved to be relevant, since thirst is the second greatest discomfort observed among critically ill patients^(8,25)^, highlighting the complexity and multifactorial nature of this symptom^(15)^.

The introduction of a photographic scale to assess oral mucosa hydration has a complementary nature, as it facilitates the identification of signs of oral mucosa dehydration, particularly in intensive care patients^(14)^. Image-based scales are a viable method for assessing hospitalized patients, as they allow the identification and classification of symptoms presented. However, further studies related to the topic and its applicability in different populations and contexts are needed.

In clinical practice, oral cavity dehydration is commonly used as one of the defining characteristics of thirst, but it is also a major factor associated with the genesis of thirst^(15)^. Thick saliva, highly present in ICU patients, was also observed in 52% of patients in the immediate postoperative period^(26)^, in which the symptom was first observed. Dry throat and dry lips, elevated in critically ill patients, were also observed as highly prevalent in postoperative patients, with 72% and 82.7%, respectively^(13)^. The same study presents characteristics such as constant swallowing of saliva (70%) and desire to drink water (72.7%) in patients in the immediate postoperative period with lower, but similar, prevalences to those observed in the ICU, evidencing the factors generating thirst common to both sectors. It was noted that all study participants, even if they verbalized the absence of thirst, presented at least one defining characteristic, as in research conducted in the Surgical Center^(13)^. The defining characteristics observed and mentioned above denote the presence of non-homeostatic thirst, usually less valued than homeostatic thirst in ICU clinical environments.

The manifestation of signs of thirst is highly prevalent in critically ill patients, although it is rarely noticed by the healthcare team. In view of this, the need for contact with the concept of thirst and its characteristics by professionals who provide care to this population is once again emphasized. The inclusion in NANDA-I of a diagnosis for thirst, with the definition of the symptom and its characteristics^(13)^, will allow this approach and will offer support to nursing conducts for thirst management.

The association of instruments for identifying and assessing thirst dimensions proposes a broad approach to the symptom by the nursing team. The EDESP, when associated with the VNS, used to measure thirst intensity, highlights the proportionality between self-perception and the discomfort felt by patients, showing the subjectivity of the symptom, as observed in patients in the immediate postoperative period^(24)^ and critically ill patients. The exacerbated time of oral fluid restriction indicates a direct influence not only on thirst intensity, but also on discomfort intensity felt by patients in the ICU, as verified by the EDESP. This scale also made it possible to conclude that the long stay of patients in specialized beds and, consequently, prolonged exposure to pharmacotherapies and intra-hospital procedures^(4)^ lead to an increase in their score.

Using the image scale in critically ill patients highlighted the complexity and multifactorial nature of the symptom^(15)^. A proportional increase in thirst intensity was observed with the scores contemplated by the Objective Oral Mucosa Scale, showing that this scale identifies characteristics of thirst present in the oral cavity that, often, may not be noticed by the patients themselves, indicating that the association of tools increases the benefits to the assisted population.

Regarding the joint use of the main scales of this study, it is important to note that both are complementary: the score obtained by EDESP is high, although the score obtained by the Objective Oral Mucosa Scale is low, suggesting that patients’ self-perception is revealed first and the characteristics observed in the oral cavity manifest themselves later. Thus, the scales cover different but complementary dimensions of thirst.

As far as research has indicated, no other studies have been found that used the Objective Oral Mucosa Scale or even the EDESP in ICU patients and correlated them with thirst presence in this population. Therefore, it is important to note and suggest that further research be carried out within the intensive care setting to expand the data on thirst in ICU patients and its related factors.

Although no significant correlations were observed between thirst presence and drug therapies in the present study, research has shown that opioid analgesics, along with diuretic medications, are the main predictors of thirst and xerostomia among critically ill patients^(7,8,9)^. More extensive studies are needed to delve deeper into this issue.

### Study limitations

The limitations identified were the non-probabilistic sample, limiting the generalization of the data, the subjectivity of the Objective Oral Mucosa Scale, which was used by a single researcher, without peer evaluation, and the lack of studies containing the scale for comparison.

### Contributions to health, nursing or public policy

The study identified thirst, its discomforts and associated factors in critically ill patients using the VNS and EDESP, scales developed and used primarily with the surgical population. The association of these instruments with a photographic scale aimed at analyzing oral cavity dehydration in ICU patients, allows a broader approach to an undervalued symptom. Using these scales in clinical practice may contribute to the intentional identification of thirst by the health team as well as provide support for safely conducting care for this group of patients, with the aim of reducing the presence and intensity of this symptom.

## CONCLUSIONS

Thirst prevalence and intensity were high, with the desire to drink water and dry lips being the greatest discomforts experienced by ICU patients. Thick saliva, constant swallowing of saliva, desire to drink water, dry lips and dry throat were the main defining characteristics of thirst. The photographic scale used identified dry lips and moist mouth with a score of 2 as the most predominant signs. The statistically significant and positive correlations reflected the complexity and multifactorial nature of thirst, demonstrating the viability of using several tools together to identify the symptom among hospitalized patients, with a special focus on the care of critically ill patients.

## Data Availability

https://doi.org/10.48331/scielodata.OHWTHC
