# Supplementary material for: Thirst in critical patients and its associated factors
Source: Rev Bras Enferm. 2025 Mar 10;78(1):e20240064. doi: 10.1590/0034-7167-2024-0064 (PMC11893054; doi:10.1590/0034-7167-2024-0064)
Supplement: Translation

# Translation: Sede em pacientes críticos e seus fatores associados

**DOI:** 10.1590/0034-7167-2024-0064pt

**Authors:** Laura Vicentim Berbert, Isadora Pierotti, Leonel Alves do Nascimento, Isabela Bossi Faleiros, Meiriane Pizani Scobare de Oliveira, Rafael Alexandre Biz, Rafaela Vieira Jorge, Lígia Fahl Fonseca

**Affiliations:** I Universidade Estadual de Londrina. Londrina, Paraná, Brasil

**Keywords:** Sede, Unidades de Terapia Intensiva, Cuidados de Enfermagem, Conforto do Paciente, Cuidados Críticos

## Abstract

**Objetivos:**

avaliar prevalência, intensidade, desconforto, características definidoras da sede e sinais de hidratação da mucosa oral em pacientes de Unidade de Terapia Intensiva.

**Métodos:**

estudo quantitativo e analítico, realizado em hospital terciário em seis das sete Unidades de Terapia Intensiva, com amostra de 60 pacientes. Variáveis relacionadas à sede foram analisadas conforme sua natureza.

**Resultados:**

a prevalência da sede foi de 76,7%, intensidade média de 7,2. O principal escore da *Objective Oral Mucosa Scale* foi 2 (61,7%), correspondendo a lábios secos e boca úmida. Características definidoras prevalentes foram saliva grossa (80%), constante deglutição de saliva (76,7%), vontade de beber água (75%), lábios ressecados (73,3%) e garganta seca (70%). Identificaram-se correlações positivas entre as escalas e intensidade da sede, tempo de restrição hídrica e internação.

**Conclusões:**

as correlações estatisticamente significantes traduzem a complexidade e multifatoriedade da sede, demonstrando a necessidade de utilização de instrumentos de identificação e mensuração na população crítica.

## INTRODUÇÃO

A sede, definida como a busca pela ingestão de água, advém da necessidade fisiológica do organismo de restabelecer seu equilíbrio hidroeletrolítico. Fisiologicamente, pode ser dividida em homeostática, aquela que integra funções orgânicas e osmóticas, com influência direta na volemia, e não homeostática, que aborda aspectos relacionados à sede, como características da ingesta hídrica e alimentar, hábitos de vida individuais e particularmente estímulos deflagrados pelo ressecamento da cavidade oral^(1)^.

No ambiente hospitalar, diversos fatores se configuram como estressores e podem resultar na gênese da sede. Entre eles, estão procedimentos cirúrgicos que requerem restrição hídrica, modalidades anestésicas adotadas, intubação orotraqueal, risco para hemorragias, entre outros^(2)^, como o processo de adoecimento e as inseguranças que permeiam esse momento^(3)^.

Esse desconforto também atinge pacientes críticos. Nas Unidades deTerapia Intensiva (UTI), a sede se apresenta com elevada prevalência, chegando a 68,9%, sendo categorizada como leve (30,9%), moderada (42,9%) e severa (26,2%)^(4)^. Os estudos específicos para tal população ainda são recentes, e a atenção a esse desconforto deve integrar os cuidados assistenciais^(5)^, uma vez que pacientes desse grupo estão suscetíveis a fatores agravantes, como maior desequilíbrio hídrico, terapias medicamentosas marcadas principalmente pelo uso de anticolinérgicos^(6)^, opioides^(7,8,9)^, anti-hipertensivos^(6)^, antidepressivos^(10)^, ventilação mecânica (VM) ^(5)^ e restrições hídricas e alimentares^(11)^.

A combinação de uso de dispositivos endotraqueais e medicações sedativas, procedimentos comuns em UTIs, contribui para que muitos pacientes fiquem inconscientes e impossibilitados de verbalizar espontaneamente sua sede e o desconforto causado por ela^(12)^. Nos pacientes críticos, portanto, o autorrelato de um sintoma subjetivo, considerado padrão-ouro para sua identificação e mensuração, fica prejudicado. A baixa frequência de verbalização de sede sugere a necessidade de atenção especial da equipe de saúde, principalmente dos enfermeiros, aos sintomas característicos de sede e encorajamento dos pacientes que a verbalizem, para que haja correta identificação e manejo do sintoma. Os profissionais de saúde devem buscar sanar desconfortos para além dos já conhecidos em UTI, bem como realizar seu registro formal e legal para que haja continuidade do cuidado^(5,6)^. Entretanto, o número de ferramentas criadas exclusivamente para o manejo da sede da população crítica é escasso, e, muitas vezes, não são validadas por especialistas^(7)^.

Na busca pela conscientização e manejo correto da sede por parte da equipe multiprofissional, surgiu a necessidade de elaborar a proposta de um diagnóstico para ser incluído em taxonomias diagnósticas, como a NANDA-I, originalmente desenvolvido como um diagnóstico de sede perioperatória, voltado sobretudo para a população cirúrgica. Estruturada nas normas do instrumento, a proposta diagnóstica traz os principais fatores etiológicos de sede e seus fatores relacionados, com destaque para as características definidoras: lábios ressecados; vontade de beber água; constante deglutição de saliva; garganta seca; saliva grossa; língua grossa; gosto ruim na boca; e relato do cuidador. A avaliação das características definidoras da sede permite complementar a identificação de sinais por parte da equipe de enfermagem, tendo aplicabilidade para uso com pacientes críticos em UTI^(13)^.

A *Objective Oral Mucosa Scale* é uma escala fotográfica chinesa desenvolvida para avaliar a desidratação da cavidade oral. O uso da escala possibilitou reconhecer a xerostomia como característica da sede de maior prevalência na população crítica, revelando ser um instrumento útil na utilização de pacotes de intervenção de alívio da sede previamente testados e aprovados^(14)^.

A sede é complexa e multifatorial^(15)^, portanto, não se deve limitar sua avaliação apenas à intensidade; é preciso considerar também os desconfortos que ela causa. Neste sentido, foi desenvolvida a Escala de Desconforto da Sede Perioperatória (EDESP), uma escala do tipo Likert que avalia sete atributos relacionados, tendo altos índices de confiabilidade e fidedignidade^(16)^.

Considerando o elevado número de pacientes em UTI que experienciam a sede, a escassez de estudos realizados com essa população e a dificuldade de realizar o manejo da sede neste grupo de pacientes devido às especificidades e criticidade inerentes a eles, o presente estudo se faz necessário para maior valorização da sede e suas características definidoras. Adicionalmente, escalas que permitem a fácil identificação dos sinais característicos da sede podem ser ferramentas úteis às equipes de saúde em seu cuidado intencional desse sintoma.

## OBJETIVOS

Avaliar a prevalência, intensidade, desconfortos, características definidoras da sede e sinais de hidratação da mucosa oral em pacientes de UTI e fatores relacionados.

## MÉTODOS

### Aspectos éticos

Este trabalho faz parte de um projeto maior denominado “Efeitos do picolé mentolado sobre secreção de vasopressina, osmolaridade, intensidade, desconforto da sede e desidratação oral: Ensaio Clínico Randomizado”, com aprovação pelo Comitê de Ética em Pesquisa. Para realização desta pesquisa, foi necessária a realização de uma emenda ao parecer anterior. O estudo foi conduzido de acordo com as diretrizes de ética nacionais e internacionais, sendo aprovado pelo Comitê de Ética em Pesquisa da Universidade Estadual de Londrina, cujo parecer está anexado à presente submissão. O Termo de Consentimento Livre e Esclarecido foi obtido de todos os indivíduos envolvidos no estudo por meio escrito.

### Desenho, período e local do estudo

Estudo quantitativo, observacional, transversal e analítico, realizado nas UTIs de um hospital universitário terciário do estado do Paraná. Trata-se de hospital público à disposição do Sistema Único de Saúde (SUS), que abrange áreas de ensino e pesquisa, com 419 leitos. Conta com sete UTIs para atendimento adulto, com 64 leitos especializados, contemplando pacientes cirúrgicos, clínicos, em isolamento, de longa permanência e alta dependência, sendo uma das unidades especializadas em queimaduras. A coleta de dados ocorreu em seis das sete UTIs existentes no hospital, excluindo-se a Unidade de Tratamento de Queimados, no período entre setembro e dezembro de 2022. Para o presente estudo, foi utilizado o *Strengthening the Reporting of Observational studies in Epidemiology* (STROBE) para nortear a metodologia.

### População ou amostra; critérios de inclusão e exclusão

A amostra foi não probabilística, totalizando 60 pacientes, e seguiu os critérios de inclusão que abrangeram ter idade acima de 18 anos, estar orientado em tempo e espaço, perguntando diretamente ao paciente “Qual seu nome? Onde o(a) senhor(a) está neste momento? Que dia é hoje?”, conforme protocolo *Montreal Cognitive Assessment* (MoCa)^(17)^, pacientes em restrição hídrica via oral e capacidade de verbalização da sede de forma espontânea ou quando perguntados. Os critérios de exclusão compreenderam os pacientes em uso de VM e/ou traqueostomizados, devido ao uso de medicamentos que impedem a plena orientação em tempo e espaço.

### Protocolo do estudo

Utilizou-se um roteiro com aspectos sobre a internação, presença e justificativa da restrição hídrica via oral e uso de medicamentos. A presença e a intensidade da sede foram avaliadas por meio da Escala Verbal Numérica (EVN), com amplitude de zero a dez, sendo zero nada de sede e dez a pior sede. Os desconfortos da sede foram avaliados por meio da EDESP, que avalia os atributos por observação direta ou via relato do paciente, como boca seca, lábios ressecados, língua grossa, saliva grossa, garganta seca, gosto ruim na boca e vontade de beber água, com pontuação máxima de 14 pontos, sendo este o maior grau de sede apresentado pelo paciente. Essa escala apresenta índices de confiabilidade e fidedignidade com alfa de Cronbach de 0,91^(16)^. Avaliaram-se também as características definidoras da sede previamente mencionadas^(13)^.

A desidratação da mucosa oral foi identificada por meio da escala fotográfica *Objective Oral Mucosa Scale,* que apresenta quatro escores: 1 = lábios e boca úmidos; 2 = lábios secos e boca úmida; 3 = lábios e boca secos; e 4 = lábios rachados e boca seca. Sua confiabilidade e coeficiente de correlação interobservador foi de 0,89^(14)^.

Os dados clínicos foram extraídos das informações presentes nos prontuários. Por último, comparava-se a *Objective Oral Mucosa Scale,* posicionada ao lado da cavidade oral do paciente, fazendo a identificação visualmente a anotando o escore no instrumento de pesquisa.

A pesquisadora elaborou o roteiro, que foi submetido à análise aparente por dois especialistas no tema sede. Aplicou-se teste-piloto com cinco pacientes; não houve necessidade de realizar alterações no instrumento; e os dados não foram incluídos na amostra.

### Análise dos resultados e estatística

As variáveis dependentes do estudo foram presença e intensidade da sede, sinais de hidratação da *Objective Oral Mucosa Scale* e características definidoras da sede. As variáveis independentes foram os dados sociodemográficos e clínicos, como sexo, idade, tempo de internação na UTI, motivo da internação, tempo de restrição hídrica via oral, uso de medicações e uso prévio de dispositivos para VM.

Os dados foram estruturados em planilha *Microsoft Excel 2019*® validados por dupla digitação e analisados com auxílio do programa estatístico *Jamovi Version 2.3.* Para análise dos dados, utilizou-se análise descritiva, com apresentação das variáveis em frequências absolutas e relativas, assim como medidas de tendência central e de dispersão. Aplicou-se o teste de Shapiro-Wilk para avaliar se os dados aderiram à normalidade. Para a correlação, utilizaram-se o teste de Spearman, para as variáveis numéricas, e o teste de associação qui-quadrado (X^2^), para as variáveis categóricas, ambos com nível de significância 0,05. Os testes que utilizaram Spearman tiveram suas forças de correlação interpretadas da seguinte maneira: fracas ou pequenas (valores observados entre 0,1 e 0,3 ou -0,1 e -0,3); moderadas ou médias (valores observados entre 0,3 e 0,5 ou -0,3 e -0,5); e fortes (valores observados entre 0,5 e 1,0 ou -0,5 e -1,0)^(18)^.

## RESULTADOS

Os participantes da pesquisa apresentaram predominância do sexo masculino (65%) e idade média de 54,6 anos (±16,6). A clínica mais prevalente foi a cardiologia (36,7%), e a média de internação correspondeu a 11,2 dias (±22,3). A média do tempo de restrição hídrica via oral foi de 37,1 horas (±79,2), com mínimo de uma hora e máximo de 432 horas. Durante a internação, prevaleceu o uso de medicamentos Opioides (53,3%). Os dados de caracterização dos participantes estão presentes na Tabela 1.

**Tabela 1:** Dados sociodemográficos e clínicos dos participantes da coleta de dados (N=60), Londrina, Paraná, Brasil, 2022

Variáveis		n	%
Sexo			
Masculino		39	65,0
Feminino		21	35,0
Clínica			
Cardiologia		22	36,7
Neurocirurgia		08	13,3
Gastroenterologia		07	11,7
Cirurgia do aparelho digestivo		05	08,3
Outras		18	30,1
Dispositivos de ingestão nutricional			
Cateter nasoenteral		06	10,0
Nutrição parenteral		01	01,7
Jejum completo sem necessidade de dispositivos		53	88,3
Motivo para necessidade da restrição hídrica via oral			
Exames		07	11,7
Pré-operatório		23	38,3
Pós-operatório		21	35,0
Outros		09	15,0
Medicamentos em uso			
Opioides		32	53,3
Antidepressivos		14	23,3
Anti-colinérgicos		06	10,0
Anti-hipertensivos		28	46,7
Uso de ventilação mecânica prévia durante a internação			
Sim		18	30,0
Não		42	70,0
**Variáveis contínuas**	**Média** (±)	**Mediana (Mínimo-Máximo)**	Valor de p*
Idade	54,6 (16,6)	59,0 (19-80)	0,015
Dias de internação	11,2 (22,3)	05,0 (01-157)	<0,001
Tempo de restrição hídrica via oral (horas)	37,1 (79,2)	12 (01-432)	<0,001

* *Valor de p referente ao teste de normalidade Shapiro-Wilk.*

A prevalência da sede foi de 76,7% (46/60). A intensidade média da sede foi de 7,2, mensurada por meio da EVN, com pontuação de zero a dez, portanto mostrando uma intensidade elevada. Em relação à EDESP, ela possui pontuação de zero a 14, sendo a pontuação média de desconforto da sede avaliada de 7,3 pontos, refletindo que a sede, para além de intensa, também é altamente desconfortável para o paciente crítico. O principal escore observado pela *Objective Oral Mucosa Scale* foi o de 2 (61,7%), correspondendo a lábios secos e boca úmida, sendo o escore 4 o único a não ser identificado. As variáveis presença de sede, intensidade, desconfortos da sede, mensuradas por meio da EDESP e escore da *Objective Oral Mucosa Scale,* estão na Tabela 2.

**Tabela 2:** Intensidade, desconfortos da sede (EDESP) e Objective Oral Mucosa Scale nos participantes da coleta de dados (N=60), Londrina, Paraná, Brasil, 2022

Variáveis	Média (±)	Mediana (Mínimo-Máximo)	Valor de p*
Intensidade da sede	07,2 (02,3)	08 (2-10)	0,020
Pontuação da EDESP**	07,4 (04,2)	07 (0-14)	<0,001
*Objective Oral Mucosa Scale*	01,9 (0,6)	02 (1-3)	
*Objective Oral Mucosa Scale*	**n**	**%**	
Escore 1	15	25,0	
Escore 2	37	61,7	
Escore 3	08	13,3	
Escore 4	00	0,0	
**EDESP**	**Nada incomodado n (%)**	**Um pouco incomodado n (%)**	**Muito incomodado n (%)**
Minha boca está seca	17 (28,3)	18 (30,0)	25 (41,7)
Meus lábios estão ressecados	21 (35,0)	13 (21,7)	26 (43,3)
Minha língua está grossa	28 (46,7)	12 (20,0)	20 (33,3)
Minha saliva está grossa	17 (28,3)	18 (30,0)	25 (41,7)
Minha garganta está seca	21 (35,0)	14 (23,3)	25 (41,7)
Sinto um gosto ruim na boca	28 (46,7)	13 (21,7)	19 (31,7)
Tenho vontade de beber água	15 (25,0)	16 (26,7)	29 (48,3)

*Valor de p referente ao teste de normalidade Shapiro-Wilk; **EDESP - Escala de Desconfortos da Sede Perioperatória.

Entre as características definidoras da sede, as mais prevalentes foram saliva grossa (80%,), constante deglutição de saliva (76,7%), vontade de beber água (75%), lábios ressecados (73,3%) e garganta seca (70%). Todas as características definidoras da sede estiveram presentes entre os pacientes e estão apresentadas na Tabela 3.

**Tabela 3:** Características definidoras da sede dos participantes da coleta de dados (N=60), Londrina, Paraná, Brasil, 2022

CARACTERÍSTICAS DEFINIDORAS	SIM n (%)	NÃO n (%)
Garganta seca	42 (70,0)	18 (30,0)
Lábios ressecados	44 (73,3)	16 (26,7)
Saliva grossa	48 (80,0)	12 (20,0)
Língua grossa	39 (65,0)	21 (35,0)
Constante deglutição de saliva	46 (76,7)	14 (23,3)
Vontade de beber água	45 (75,0)	15 (25,0)
Gosto ruim na boca	33 (55,0)	27 (45,0)
Relato do cuidador	02 (3,3)	58 (96,7)

Houve correlações positivas moderadas entre: a EDESP e *Objective Oral Mucosa Scale;* o tempo de restrição hídrica via oral e a EDESP; o tempo de internação e a EDESP; a intensidade da sede e a EDESP; e a intensidade da sede e a *Objective Oral Mucosa Scale.* As correlações podem ser observadas no Quadro 1.

**Quadro 1:** Coeficientes de correlação de Spearman (r_s_) entre variáveis relacionadas à sede (N=60), Londrina, Paraná, Brasil, 2022

Variáveis	EDESP*	Intensidade da sede	Objective Oral Mucosa Scale	Restrição hídrica via oral	Dias de internação
EDESP	-				
Intensidade da sede	r_s=_0,473 p<0,001	-			
*Objective Oral Mucosa Scale*	r_s=_0,364 p 0,004	r_s=_0,424 p 0,003	*-*		
Restrição hídrica via oral	r_s=_0,378 p 0,003	r_s=_0,303 p 0,041	r_s=_0,230 p 0,077	-	
Dias de internação	r_s=_0,332 p 0,009	r_s=_-0,029 p 0,846	r_s=_0,094 p 0,474	r_s=_-0,046 p 0,729	-

* *EDESP - Escala de Desconforto da Sede Perioperatória.*

A análise de variáveis categóricas (medicamentos, uso de VM e presença da sede) não encontrou dados significativos, observados na Tabela 4.

**Tabela 4:** Variáveis categóricas correlacionadas à presença de sede, Londrina, Paraná, Brasil, 2022

Variáveis	X^2^	Valor de p*
Medicamentos Opioides	3,29	0,070
Medicamentos anti-colinérgicos	0,839	0,360
Medicamentos antidepressivos	0,0284	0,866
Medicamentos anti-hipertensivos	2,28	0,131
Uso de ventilação mecânica prévia	3,23	0,072

* *teste qui-quadrado (X^2^) com correção de continuidade.*

## DISCUSSÃO

Este estudo traz um panorama de avaliação da sede em pacientes críticos e seus fatores associados dentro de um cenário complexo, o de pacientes em UTI, tema insuficientemente explorado pela literatura. Não obstante, a prevalência da sede foi avaliada por meio de uma escala originalmente estruturada para pacientes em pós-operatório imediato, além da intensidade, suas características definidoras e como essas geram desconforto no paciente crítico. Além disso, complementou-se a avaliação por meio de uma escala fotográfica voltada para a hidratação da mucosa oral, uma das principais fontes de manifestação da sede.

O campo dos cuidados intensivos comumente aborda populações de pacientes com quadros clínicos delicados e que necessitam de internações de longa permanência em setores especializados, uma vez que os recursos terapêuticos podem incluir abordagens cirúrgicas e uso de VM^(5,8)^. A cultura do jejum prolongado, característica marcante do hospital onde se desenvolveu a pesquisa, opõe-se ao tempo de restrição hídrica via oral preconizado pela *American Society of Anesthesiologists* (ASA), que corresponde a até duas horas para líquidos claros^(19)^ para pacientes cirúrgicos em pré-operatório.

Ainda que o uso de VM pelos pacientes do presente estudo tenha se apresentado baixo, dispositivos oro e endotraqueais se mostraram determinantes para a gênese da sede^(5)^. Esses atributos são considerados fatores predisponentes para a sede, sendo este sintoma um estressor importante, principalmente para pacientes cardiopatas^(20)^.

A sede mostra-se elevada e intensa entre os pacientes críticos, com prevalência observada acima dos 75%, sendo expressa, sobretudo, pela sede severa por 44,9% dos pacientes^(6)^. A elevada intensidade do sintoma no presente estudo reforça essa população como de alto risco para o desenvolvimento de sede, devido a inúmeros fatores desencadeantes, como o uso de Opioides e diuréticos, níveis séricos de sódio e glicose, capacidade de ingestão oral, jejum, osmolaridade e VM com ressecamento da cavidade oral^(4)^. Outros estudos trazem o papel da administração de fármacos especializados, dispositivos e abordagens invasivas como preditores da sede^(1,6,21)^. Desta forma, observa-se que tanto a sede homeostática quanto a sede não homeostática foram ativadas por mecanismos fisiopatológicos e ambientais^(1)^.

Uma vez que a sede se caracteriza como um desconforto intenso e que resulta em sentimentos negativos ao paciente^(8,22)^, faz-se necessário o olhar intencional da equipe de enfermagem para que o desconforto seja identificado e manejado corretamente^(5)^. Estratégias que deflagram mecanismos de saciedade pré-absortiva por meio de temperaturas frias e mentol têm se mostrado efetivas para aliviar significativamente a sede e o desconforto de pacientes em terapia intensiva. Medidas como *sprays* mentolados conferem alívio com baixo volume e, consequentemente, maior segurança para o paciente crítico em restrição hídrica^(23)^.

A sede pode ser identificada pela aplicação de escalas específicas. A EDESP, originalmente projetada para atender pacientes em pós-operatório imediato^(16)^, mostrou-se uma escala versátil. Capaz de identificar diversos fatores de desconforto, ela não se limita à sua população-alvo, uma vez que os setores crítico e cirúrgico compartilham aspectos comuns, como o jejum, as medicações, a necessidade de VM e a complexidade de seus pacientes^(4,5)^. A utilização dessa escala na UTI trouxe resultados paralelos aos de pesquisa com pacientes cirúrgicos, apresentando intensidade média de desconforto de 7,3 e o atributo “tenho vontade de beber água” como o mais incômodo^(24)^. A aplicabilidade de uma escala direcionada para os desconfortos na rotina de avaliação de pacientes de UTI mostrou-se relevante, já que a sede é o segundo maior desconforto observado entre pacientes críticos^(8,25)^, ressaltando a complexidade e a multifatoriedade desse sintoma^(15)^.

A introdução de uma escala fotográfica para avaliação da hidratação da mucosa oral apresenta um caráter complementar, por facilitar a identificação de sinais de desidratação da mucosa oral, particularmente em pacientes em terapia intensiva^(14)^. Escalas imagéticas são um método viável para avaliação de pacientes hospitalizados, pois permite identificar e classificar os sintomas apresentados. No entanto, são necessários mais estudos relacionados ao tema e sua aplicabilidade em diferentes populações e contextos.

Na prática clínica, a desidratação da cavidade oral comumente é utilizada como uma das características definidoras da sede, todavia também é, principalmente, fator associado para a gênese da sede^(15)^. A característica saliva grossa, altamente presente em pacientes de UTI, também foi constatada em 52% de pacientes em pós-operatório imediato^(26)^, em que o sintoma foi primeiramente observado. As características garganta seca e lábios ressecados, elevadas em pacientes críticos, também foram observadas como altamente prevalentes em pacientes pós-operatórios, com 72% e 82,7%, respectivamente^(13)^. O mesmo estudo traz características como constante deglutição de saliva (70%) e vontade de beber água (72,7%) em pacientes em pós-operatório imediato com prevalências menores, porém semelhantes, às observadas na UTI, evidenciando os fatores geradores de sede comuns a ambos os setores. Notou-se que todos os participantes do estudo, ainda que verbalizassem ausência de sede, apresentaram ao menos uma característica definidora, assim como em pesquisa conduzida em Centro Cirúrgico^(13)^. As características definidoras observadas e supracitadas denotam a presença da sede não homeostática, usualmente menos valorizada que a sede homeostática nos ambientes clínicos de UTI.

A manifestação dos sinais de sede é altamente prevalente em pacientes críticos, apesar de pouco percebida pela equipe de saúde. À vista disso, ressalta-se novamente a necessidade de contato com a sede e suas características por parte dos profissionais que prestam assistência a essa população. A inserção na NANDA-I de um diagnóstico para a sede, com a definição do sintoma e suas características^(13)^, permitirá essa aproximação e oferecerá suporte às condutas de enfermagem para o manejo da sede.

A associação de instrumentos para identificação e avaliação das dimensões da sede propõe uma ampla abordagem do sintoma pela equipe de enfermagem. A EDESP, quando associada à EVN, utilizada para mensuração da intensidade da sede, ressalta a proporcionalidade entre a autopercepção e os desconfortos sentidos pelo paciente, mostrando a subjetividade do sintoma, como se observou em pacientes em pós-operatório imediato^(24)^ e pacientes críticos. O tempo exacerbado de restrição hídrica via oral sinaliza influência direta não somente na intensidade da sede, mas também na intensidade dos desconfortos sentidos pelos pacientes em UTI, verificados pela EDESP. Tal escala também possibilitou concluir que a longa permanência dos pacientes em leitos especializados e, consequentemente, a exposição prolongada a farmacoterapias e procedimentos intra-hospitalares^(4)^ acarretam o aumento de sua pontuação.

A utilização da escala imagética em pacientes críticos evidenciou a complexidade e a multifatoriedade do sintoma^(15)^. Observou-se o aumento proporcional da intensidade da sede com os escores contemplados pela *Objective Oral Mucosa Scale,* mostrando que esta escala identifica características da sede presentes na cavidade oral que, muitas vezes, podem não ser notadas pelo próprio paciente, apontando que a associação de ferramentas amplia os benefícios à população assistida.

No que diz respeito à utilização conjunta das principais escalas deste estudo, importa dizer que ambas têm caráter de complementaridade: a pontuação obtida pela EDESP é elevada, ainda que o escore obtido pela *Objective Oral Mucosa Scale* seja baixo, sugerindo que a autopercepção do paciente se revela primeiramente e as características observadas na cavidade oral se manifestam em seguida. Dessa forma, as escalas abrangem dimensões da sede diferentes, mas complementares.

Até onde se pesquisou, não se localizaram outros estudos que utilizaram a escala fotográfica *Objective Oral Mucosa Scale* ou mesmo a EDESP em pacientes de UTI e correlacionaram com a presença de sede nessa população. Atenta-se e sugere-se, portanto, a realização de outras pesquisas dentro do cenário intensivo para ampliar os dados acerca da sede em pacientes de UTI e seus fatores relacionados.

Apesar de não terem sido observadas correlações significativas entre a presença da sede e terapias medicamentosas no presente estudo, pesquisas evidenciaram analgésicos da classe dos Opioides, juntamente com medicamentos diuréticos, como os principais preditores da sede e da xerostomia entre os pacientes críticos^(7,8,9)^. É necessária a realização de estudos mais amplos para o aprofundamento desta questão.

### Limitações do estudo

Identificaram-se como limitação amostra não probabilística, limitando a generalização dos dados, a subjetividade da *Objective Oral Mucosa Scale,* que foi utilizada por uma única pesquisadora, sem avaliação interpares, e a falta de estudos que contivessem a escala para comparação.

### Contribuições para as áreas da saúde, enfermagem ou políticas públicas

O estudo identificou a sede, seus desconfortos e fatores associados em pacientes críticos por meio da EVN e EDESP, escalas desenvolvidas e utilizadas, primeiramente, com a população cirúrgica. A associação desses instrumentos a uma escala fotográfica voltada para a análise da desidratação da cavidade oral para pacientes de UTI permite uma abordagem mais ampla de um sintoma subvalorizado. O uso dessas escalas na prática clínica poderá contribuir para a identificação intencional da sede pela equipe de saúde, bem como fornecer subsídios para conduzir com segurança os cuidados prestados a esse grupo de pacientes, com a finalidade de reduzir a presença e a intensidade desse sintoma.

## CONCLUSÕES

A prevalência e a intensidade da sede foram elevadas, sendo a vontade de beber água e estar com os lábios ressecados os maiores desconfortos experienciados pelos pacientes em UTI. Prevaleceram saliva grossa, constante deglutição de saliva, vontade de beber água, lábios ressecados e garganta seca como principais características definidoras da sede. A escala fotográfica utilizada identificou lábios secos e boca úmida por meio do escore 2 como os sinais mais predominantes. As correlações estatisticamente significantes e positivas traduziram a complexidade e a multifatoriedade da sede, apresentando a viabilidade do uso conjunto de diversas ferramentas para identificação do sintoma entre os pacientes hospitalizados, com enfoque especial no cuidado de pacientes em estado crítico.

## Data Availability

https://doi.org/10.48331/scielodata.OHWTHC
